# Inhibition of miRNA‐27b enhances neurogenesis via AMPK activation in a mouse ischemic stroke model

**DOI:** 10.1002/2211-5463.12614

**Published:** 2019-04-11

**Authors:** Zhengang Wang, Yimei Yuan, Zhaoguang Zhang, Kuiying Ding

**Affiliations:** ^1^ Department of Neurosurgery Affiliated Hospital of Weifang Medical University China; ^2^ Department of Ultrasonography Affiliated Hospital of Weifang Medical University China; ^3^ Technology Center Weifang Entry‐exit Inspection and Quarantine Bureau China

**Keywords:** AMPK, hippocampal dentate gyrus, miRNA‐27b, neurogenesis, stroke, subventricular zone

## Abstract

Stroke is a leading cause of death and disability, but treatment options remain limited. Recent studies have suggested that cerebral ischemia‐induced neurogenesis plays a vital role in post‐stroke repair. Overactivation of AMP‐activated protein kinase (AMPK), a master sensor of energy balance, has been reported to exacerbate neuron apoptosis, but the role of chronic AMPK stimulus in post‐stroke recovery remains unclear. MicroRNAs have emerged as regulators of neurogenesis and have been reported to be involved in neurological function. In this study, we verified that miR‐27b directly targets AMPK and inhibits AMPK expression. In cultured neural stem cells, miR‐27b inhibitor improved proliferation and differentiation via the AMPK signaling pathway, but did not have an obvious effect on cell viability under oxygen and glucose deprivation conditions. In a mouse middle cerebral artery occlusion model, administration of miR‐27b inhibitor significantly enhanced behavioral function recovery and spatial memory. Up‐regulation of neurogenesis was observed both in the subventricular zone and in the hippocampal dentate gyrus. Collectively, our data suggest that miR‐27b inhibition promotes recovery after ischemic stroke by regulating AMPK activity. These findings may facilitate the development of novel therapeutic strategies for stroke.

**Abbreviations**
ACC: acetyl‐CoA carboxylase
AICAR: 5‐aminoimidazole‐4‐carboxamide ribonucleotide
AMPK: AMP‐activated protein kinase
BrdU: 5‐bromo‐2′‐deoxyuridine
CBF: cerebral blood flow
DCX: doublecortin
DG: dentate gyrus
DMEM: Dulbecco's modified Eagle's medium
EBSS: Earle's Balanced Salt Solution
MCAo: middle cerebral artery occlusion
miR: microRNA
miRNA: microRNA
mTOR: mechanistic target of rapamycin
MTT: 3‐(4,5‐dimethylthiazol‐2‐yl)‐2,5‐diphenyl‐tetrazolium bromide
NeuN: neuronal nuclei
NSC: neural stem cell
OGD: oxygen–glucose deprivation
PKCζ/λ: protein kinase C ζ/λ
SVZ: subventricular zone
TTC: 2,3,5‐triphenyltetrazolium chloride
Tuj1: class III β‐tubulin
UTR: untranslated region
WT: wild‐type

Stroke is one of leading causes of death and long‐term disability worldwide 1. Ischemic stroke accounts for more than 80% of stroke, local ischemia and a hypoxic environment induce neuronal cell apoptosis, necrosis and other metabolism‐related disorders. At present, recombinant tissue plasminogen activator is the only FDA‐approved treatment for acute ischemic stroke, but a narrow therapeutic window limits its clinic usage 2. Meanwhile, there is no neuroprotective agent that has shown prospective results in clinical trials.

Brain repair processes after ischemic stroke are attracting more attention. Accumulating studies have suggested that cerebral ischemia‐induced neurogenesis 3, 4, 5, which occurs mainly in the subventricular zone (SVZ) and subgranular zone in hippocampal dentate gyrus (DG) 6, might give rise to a cure strategy for recovery of brain function 7, 8. In DG, newborn neurons are continuously produced throughout adulthood 9, 10, 11. A manipulated increase of neurogenesis in DG was reported to improve synaptic plasticity and memory 12, 13. For now, it remains a challenge to amplify this process to enhance neurological function after injury.

The role that AMP‐activated protein kinase (AMPK) plays in ischemic brain remains a matter of debate. Several studies reported that AMPK activation is detrimental, since it enhances energy‐consumptive pathways that further contribute to the death of injured neuronal cells 14, 15, 16. Meanwhile, Kuramoto *et al*. 17 suggested that AMPK leads to neuroprotection through functional modulation of the GABA_B_ receptor. Besides the effects on neural apoptosis and mortality, AMPK has been reported to play a dual role in generation of new neurons; the divergent effects result from the duration and extent of AMPK activation 18, 19. The use of low dose 5‐aminoimidazole‐4‐carboxamide ribonucleotide (AICAR; an AMPK direct activator) enhanced neurogenesis and cognition, while high dose AICAR impaired cognition and induce cell apoptosis 20.

MicroRNAs (miRNAs) are small non‐coding RNAs of ~ 20 nucleotides that act as post‐transcriptional regulators of their targets mRNAs 21. The studies of other groups have indicated that miRNAs play an important role in neuroprotection and post‐stroke recovery 22, 23, 24, 25, 26, 27. It has been demonstrated that miR‐27b was markedly reduced in numerous types of cancer and exerted anti‐tumor effects by suppressing cell proliferation 28, but its effects on neurogenesis after ischemic stroke have not been investigated.

In the present study, we confirmed the direct targeting of miR‐27b on AMPKα2, the main effective AMPKα isoform in ischemic brain 14. We down‐regulated miR‐27b expression in cultured neural stem cells (NSCs) and observed mild stimulation of AMPK. In conditions of ischemia and hypoxia, the enhancement of AMPK activity by anti‐miR‐27b improved neurogenesis and post‐stroke neurological function recovery.

## Materials and methods

### Luciferase assay

**Figure 1:**
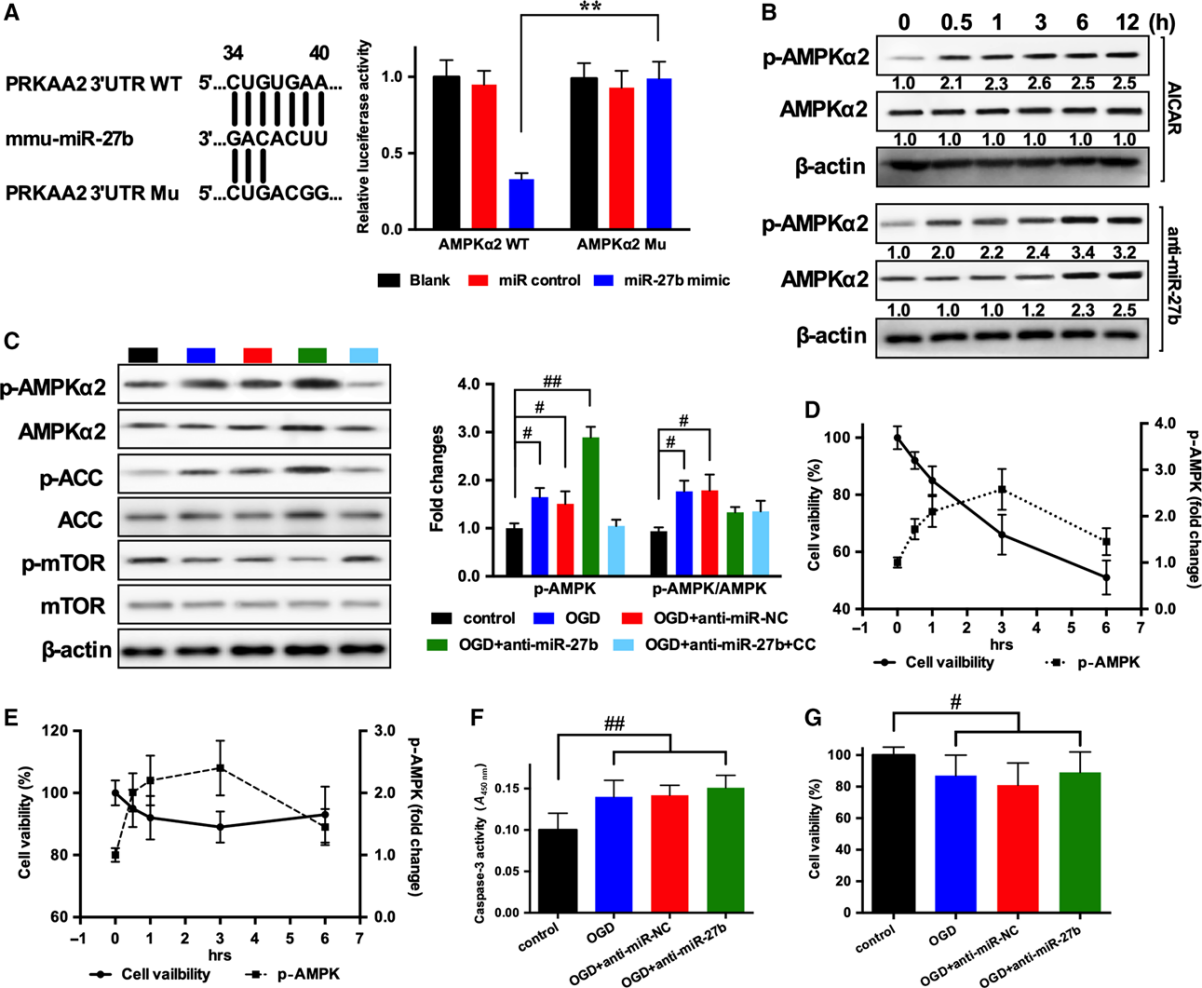
Effects of AMPK activation on neuron and NSC viability in OGD treatment. (A) Schematic diagram of miR‐27b binding site in WT AMPKα2 (*PRKAA2*) 3′UTR fragment and design of mutated sequence; and luciferase reporter assay of WT or mutant 3′UTR of *PRKAA2* with miR control or miR‐27b mimic in HEK 293T cells. (B) Time course of AMPKα2 and p‐AMPKα2 expression in NSCs treated with AICAR or transfected with anti‐miR‐27b. (C) Western blot analysis of AMPKα2, p‐AMPKα2, ACC, p‐ACC, mTOR and p‐mTOR expression and their quantitative data in NSCs transfected with miR negative control, anti‐miR‐27b, or anti‐miR‐27b with added compound C (1 μm) at 6 h. (D,E) Time courses of OGD‐induced AMPK activation and cell viability in cortical neurons (D) and NSCs (E). (F) Caspase activity tested using a Caspase‐3 Colorimetric Assay kit. (G) MTT assay was used to test the viability of NSCs treated with anti‐miR‐27b in OGD condition. *n* = 3/group. The data are expressed as mean ± SEM, **P <* *0.01 WT *vs* Mu; ^#^ *P *<* *0.05, ^##^ *P *<* *0.01 *vs* control (one‐way ANOVA followed by *post hoc* Tukey's test).

Luciferase reporter plasmid carrying wild‐type (WT) or mutant AMPKα2 gene (*PRKAA2*‐3′UTR WT/Mu) was constructed and cloned in a pEZX‐MT06 vector (Genecopoeia, Rockville, MD, USA). Point mutations were made in the miR‐27b binding site of 3′‐untranslated region (UTR) of the AMPKα2 gene (Fig. 1A). HEK293T cells (ATCC, Manassas, VA, USA) were seeded in 24‐well plate (10^5^/well) and co‐transfected with 100 ng miR‐27b mimics (UUCACAGUGGCUAAGUUCUGC; Dharmacon, Lafayette, CO, USA) or 100 ng miRNA mimic negative control (Dharmacon, cat. no.: CN‐001000‐01‐05, USA) and WT or mutant AMPKα2 (100 ng) using Lipofectamine‐2000 transfection reagent according to the manufacturer's instructions (Life Technologies, Carlsbad, CA, USA). About 48 h later, cells were lysed and luciferase activity was detected using a dual‐luciferase assay kit (Genecopoeia) and luminometer (Thermo Fisher Scientific, Waltham, MA, USA).

### Neural stem cells culture

Isolation of mouse NSCs was performed as described previously 29, 30. Mouse brains were cut sagittally and SVZ was dissociated from lateral ventricles. Tissue was gently minced and digested in 0.05% trypsin (Sigma‐Aldrich, St. Louis, MO, USA) in Dulbecco's modified Eagle's medium (DMEM; 1 mL per 100 mg tissue) at 37 °C for 1 h. Digestion was stopped with an equal volume of 0.1% trypsin inhibitor (Sigma‐Aldrich). The solution was centrifuged at 300 ***g*** for 10 min, the supernatant discarded, and the pellet suspended in proliferation medium: DMEM/F12 (Life Technologies) supplemented with epidermal growth factor (20 ng·mL^−1^; R&D Systems, Minneapolis, MN, USA), fibroblast growth factor (10 ng·mL^−1^; R&D System), N2 supplement (1%; Thermo Fisher Scientific), B27 supplement (3%; Thermo Fisher Scientific) and glutamax (1% Thermo Fisher Scientific). The medium was half changed twice a week. When spherical structures formed, the spheres were mechanically dissociated to a single cell suspension and seeded to 1% laminin‐coated plates. The next day, NSCs were transfected with 10 μm miR‐27b inhibitor or 10 μm miR inhibitor negative control (anti‐miR‐27b inhibitor, 5′‐GCAGAACUUAGCCACUGUGAA‐3′; anti‐miR‐NC, 5′‐CAGUACUUUUGUGUAGUACAA‐3′, synthesized by RiboBio Co., Guangzhou, China) using Lipofectamine‐2000 31. About 48 h later, NSCs could be used for cell viability assay, differentiation induction and immunostaining.

### Cortical neuronal culture

Cortical neurons were isolated from 18‐day‐old male C57BL/6 mice as described 32. Neurons were dissociated with 0.125% trypsin and pre‐purified by density gradient. Neurons were seeded at a density of ~ 2500 cells·mm^−2^ and cultured using DMEM containing 20% FBS. On the next day, the medium was changed to neuron‐basal medium supplemented with 2% B27 (R&D Systems). Neurons were cultured for another 3 days and used for cell viability assay.

### Oxygen–glucose deprivation and cell viability assay

Cortical neurons and NSCs were washed with PBS three times, then placed in glucose‐free Earle's Balanced Salt Solution (EBSS) buffer and stored in an anoxic chamber filled with 95% N_2_ and 5% CO_2_ for 6 h 33. For the control, cells remained in normoxic conditions. The cell survival rates were determined using the 3‐(4,5‐dimethylthiazol‐2‐yl)‐2,5‐diphenyl‐tetrazolium bromide (MTT) assay. MTT was added into the medium at a final concentration of 0.5 mg·mL^−1^ and incubated at 37 °C for 4 h. The MTT formazan was dissolved in DMSO and the absorbance was measured at 490 nm. Caspase‐3 activity was tested using a Caspase‐3 Colorimetric Assay kit (Abcam, Cambridge, MA, USA).

### Immunofluorescence staining and quantification

Neural stem cells were transfected with anti‐miR‐27b or anti‐miR‐NC and cultured for another 5 days. To label the dividing cells, 5 μm 5‐bromo‐2′‐deoxyuridine (BrdU; Sigma‐Aldrich) was added to the proliferation medium and the cells were cultured for another 8 h. The cells were then fixed and incubated with rabbit anti‐BrdU (1 : 2000; Abcam) overnight at 4 °C and then incubated with goat anti‐rabbit IgG (FITC, 1 : 300; Abcam) for 2 h. The slides were washed and mounted using mounting medium (Vector Laboratories, Burlingame, CA, USA) containing 4′,6‐diamidino‐2‐phenylindole for nucleus staining. To detect production of neurons, NSCs were transfected as described above and cultured in differentiation medium (the proliferation medium supplemented with 5% FBS, without any growth factors) for 5 days, then cells were fixed and blocked and incubated with mouse anti‐neuron‐specific class III β‐tubulin (Tuj1, 1 : 300; Abcam) overnight at 4 °C. The next day, cells were washed and incubated with goat anti‐mouse IgG (FITC, 1 : 300; Abcam) secondary antibody for 2 h. The slides were washed and mounted as above.

For quantification, BrdU^+^ or Tuj1^+^ and total cell numbers were counted in 10 random view fields/well and three wells/group under a ×20 objective lens. The results were presented as the percentage of immunoreactive cells.

To determine neurogenesis *in vivo*, coronal sections of brains were stained with the following markers: rabbit anti‐doublecortin (DCX; 1 : 100; Abcam), rabbit anti‐neuronal nuclei (NeuN; 1 : 100; Abcam). Images were taken under a ×40 objective lens, and five random view fields were chosen for each section. One‐in‐ten series of sections were stained for each animal.

### Animal experimental procedures

All experimental procedures were carried out in accordance with the *Care and Use of Laboratory Animals Guide* from the Ministry of Public Health of China and approved by the medical ethics committee of Weifang Medical University and Shandong University. Male C57BL/6J mice (~ 10 weeks, 18–22 g, specific pathogen free) were purchased from Animal Centre of Shandong University. The animals were acclimatized for a week before surgery. On day 0, all mice underwent functional tests and performed permanent middle cerebral artery occlusion (MCAo; sham group only exposed arteries, no sutures were inserted) 31, 34, 35. Briefly, animals were anesthetized and the common carotid artery, external carotid artery and internal carotid artery were exposed on the right side. An 8‐0 nylon filament was inserted from the external carotid artery into the lumen of the internal carotid artery. Two hours later, the motor tests were re‐done in a modified neurological severity score (mNSS) test (Table 1). The animals were randomly divided and received scores of 3–5 in the following three groups: MCAo, antagomir negative control (200 μg·kg^−1^, antagomir‐NC; GenePharma, Shanghai, China) and antagomir‐27b (200 μg·kg^−1^; GenePharma); *n* = 15 for each group. Antagomir negative control or antagomir‐27b was i.v. injected and the same dose of antagomir was injected on days 7, 14 and 28. Cell proliferation was assessed using BrdU incorporation; five mice from each group were i.p. injected with 100 mg·kg^−1^ BrdU from day 21 to day 28 after MCAo. At the end of the study, these animals were perfused with saline and 4% paraformaldehyde, and then brains were removed for immunostaining. Five mice from each group were sacrificed and their brains removed for 2,3,5‐triphenyltetrazolium chloride (TTC) staining. The other five animals were tested for cerebral blood flow (CBF) using laser Doppler flowmetry (LSFG‐ANW, Softcare, Ltd., Fukuoka, Japan). Using the same filter and image solution setting, relative blood flow in regions of interest from both the contralateral and the ipsilateral half brain was recorded and analyzed, and then mice were sacrificed and the brains removed for protein extraction and western blot analysis.

**Table 1:** Modified neurological severity score

Tests	Points
Motor tests	
Raising rat by the tail	
Flexion of forelimb	1
Flexion of hindlimb	1
Head moved > 10° to vertical axis within 30 s	1
Placing rat on the floor	
Normal walking	0
Inability to walk straight	1
Circling toward the paretic side	2
Falling down on the paretic side	3
Sensory tests	
Placing test (visual and tactile test)	1
Proprioceptive test (deep sensation, pushing the paw against the table edge to stimulate limb muscles)	1
Beam balance tests	
Balances with steady posture	0
Grasps side of beam	1
Hugs the beam and one limb falls down from the beam	2
Hugs the beam and two limbs fall down from the beam, or spins on beam (> 60 s)	3
Attempts to balance on the beam but falls off (> 40 s)	4
Attempts to balance on the beam but falls off (> 20 s)	5
Falls off: no attempt to balance or hang on to the beam (< 20 s)	6

### Behavioral tests

A modified neurological severity score test was performed before MCAo, and on days 1, 4, 7, 14 and 28 after MCAo according to a previous report 31, 36. All animals were tested for motor and sensory abilities, balance, reflex and abnormal movements. The evaluation standard is shown in Table 1; higher scores represent severer injury and the maximum score is 14.

The Morris water maze was used as described 31, 37. A white swimming pool 150 cm in diameter was filled with water to a depth of 30 cm. Pool temperature was maintained at 25 ± 0.5 °C. The escape platform was placed in the center of one quadrant of the pool, 1 cm below the surface of the water. Mice were allowed to find the submerged platform within 90 s. If they failed, the mice were placed on the platform for another 15 s. On each training day, each mouse was released from four different start points. The training was repeated for five consecutive days. The escape latency and the time spent in the quadrant with the platform were recorded .

### Western blot analysis

Samples were ground and lysed in RIPA buffer containing 1% protease inhibitor and 1% phosphatase inhibitor (Cell Signaling Technology, Beverly, MA, USA) and centrifuged for 12 min at 12 000 ***g***, 4 °C to remove debris. A BCA assay was used to determine protein concentrations (Beyotime, Shanghai, China). About 50 μg total protein for each sample was separated by SDS/PAGE and transferred to PVDF membrane. Membranes were incubated with the following primary antibodies: AMPKα2 (1 : 1000; Cell Signaling Technology), phospho (p)‐AMPKα2 (Thr172, 1 : 1000; Cell Signaling Technology), acetyl‐CoA carboxylase (ACC) (1 : 1000; Cell Signaling Technology), p‐ACC (Ser79, 1 : 1000; Cell Signaling Technology), mechanistic target of rapamycin (mTOR) (1 : 1000; Cell Signaling Technology), p‐mTOR (Ser248, 1 : 1000; Cell Signaling Technology), protein kinase C ζ/λ (PKCζ/λ) (1 : 1000; Cell Signaling Technology), p‐PKCζ/λ (Thr410/403, 1 : 1000; Cell Signaling Technology), and β‐actin (1 : 1000; Abcam). Signals were visualized by enhanced ECL substrate (Beyotime).

### Statistical analysis

All data are presented as the mean ± standard error (SE). Data analysis was performed using prism 6 (GraphPad Software, San Diego, CA, USA). ANCOVA was used to analyze functional test data; non‐parametric one‐way ANOVA followed by a *post hoc* Tukey's test was used to compare the difference in cell viability data, immunofluorescence staining data, and western blot data among groups. Statistical significance was set at *P *<* *0.05.

## Results

### MiR‐27b directly targets AMPKα2

MiR‐27b was predicted to match the 3′UTR of *PRKAA2* (the gene encoding AMPKα2) mRNA according to the gene sequence database at targetscan.org (Fig. 1A). We employed a luciferase assay to confirm the targeting relation of miR27b and AMPKα2. In cells co‐transfected with WT AMPKα2 3′UTR (AMPKα2 WT) and miR‐27b mimic, luciferase activity was significantly decreased compared to miR control or mutant AMPKα2 3′UTR (AMPKα2 Mu), which indicated that AMPKα2 is directly targeted by miR‐27b (Fig. 1A).

### The effect of AMPK activation by anti‐miR‐27b on NSCs

To test the effect of anti‐miR‐27b AMPK activation, AICAR (1 mm), Compound C (an AMPK inhibitor, 1 μm), and anti‐miR‐27b were administrated at the indicated dose and the NSC culture was exposed to oxygen–glucose deprivation (OGD). We compared the expression of p‐AMPKα2 in AICAR‐treated and anti‐miR‐27b‐transfected NSCs. AICAR enhanced p‐AMPKα2 expression starting at 0.5 h (2.1‐fold) but AMPKα2 expression was not affected. The effect of anti‐miR‐27b was detected at 6 h, and both p‐AMPKα2 and AMPKα2 were significantly increased (3.4‐fold and 2.3‐fold, respectively; Fig. 1B). At 6 h, anti‐miR‐27b increased the expression of p‐AMPKα2 and p‐ACC (a down‐stream target of AMPK in which p‐ACC level generally reflects the AMPK activity) (Fig. 1C). The AMPK–mTOR axis appears to have a role in neurodegenerative processes. The expression pattern of p‐mTOR was the converse of that of p‐AMPK, while total mTOR expression was not affected (Fig. 1C).

### The effect of AMPK activation on cell viability in OGD treatment

AMP‐activated protein kinase is known to be activated by energy deficiency. To determine the effect of activated AMPK on apoptosis, we examined the time course of AMPK activity and cell viability in OGD treatment. Both in cortical neurons and in NSCs, p‐AMPKα2 expression was significantly increased 0.5 h after OGD treatment and further enhanced at 6 h (Fig. 1D,E). OGD decreased neuron viability to 51% at 6 h (Fig. 1D). In NSCs, the time course for p‐AMPKα2 expression was similar to that in neurons, but cell viability was slightly decreased to 89% with OGD treatment (Fig. 1E). These data indicate that in a hypoxic environment, AMPK stimuli might play different roles in different cell types. Next, we prolonged the OGD to 24 h, and tested the NSC viability. In OGD‐treated groups, cell numbers were slightly increased compared to control group; anti‐miR‐NC and anti‐miR‐27b treatments exerted no obvious effects on apoptosis (Fig. 1F,G).

### AMPK activation by anti‐miR‐27b improved proliferation and differentiation of NSCs

**Figure 2:**
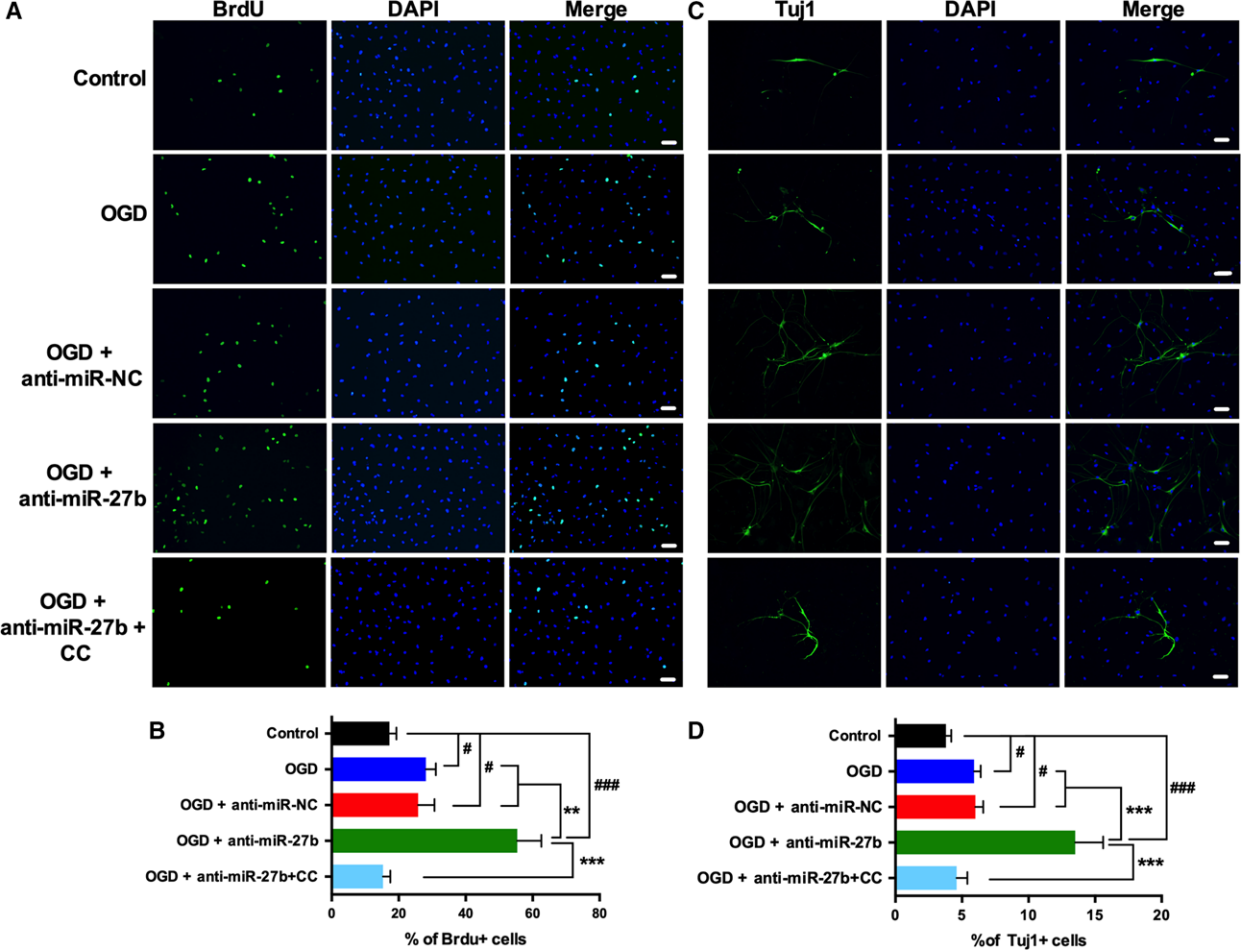
MiR‐27b is involved in neurogenesis via regulating AMPK activity. (A, B) Representative images and quantitative data of BrdU‐positive cells in NSCs treated with miR‐27b inhibitor negative control (anti‐miR‐NC), miR‐27b inhibitor, or miR‐27b inhibitor with Compound C (1 μm). (C, D) Representative images and quantitative data of Tuj1‐positive cells in NSCs treated the same way as above. *n* = 3/group. The data are expressed as mean ± SEM, ^#^ *P *<* *0.05, ^###^ *P *<* *0.001 *vs* control; **P <* *0.01, ***P <* *0.001 *vs* OGD + anti‐miR‐27b (one‐way ANOVA followed by *post hoc* Tukey's test). Scale bar = 50 μm.

NSCs cultured in growth medium were transfected with anti‐miR‐NC or anti‐miR‐27b, and another group of cells were transfected with miR‐27b inhibitor with Compound C added (1 μm). Immunofluorescence staining showed that OGD increased the percentage of BrdU‐positive cells compared with control group (28.1 ± 3.0% *vs* 17.2 ± 2.1%, *P* < 0.05) (Fig. 2A,B). Anti‐miR‐27b further up‐regulated the BrdU^+^ cell numbers by 55.4 ± 6.3%. But the effect of anti‐miR‐27b was blocked by Compound C (%BrdU^+^ cells was 15.3 ± 2.2%). Next, we tested differentiation of NSCs in all groups treated the same way as above. Except the control group, NSCs were cultured in an OGD incubator for 24 h followed by 4 days in normal conditions. The percentage of Tuj1‐positive cells was increased after OGD treatment (3.8 ± 0.4% *vs* 5.9 ± 0.5%, *P* < 0.05). Anti‐miR‐27b further enhanced the production of new neurons to 13.5 ± 2.1%. Compound C reduced the percentage Tuj1^+^ cells to 4.6 ± 0.8% (Fig. 2C,D). These data indicated that, AMPK activation by anti‐miR‐27b transfection induced NSCs proliferation and differentiated into new neurons.

### Antagomir‐27b improved neurological recovery in MCAo mice

**Figure 3:**
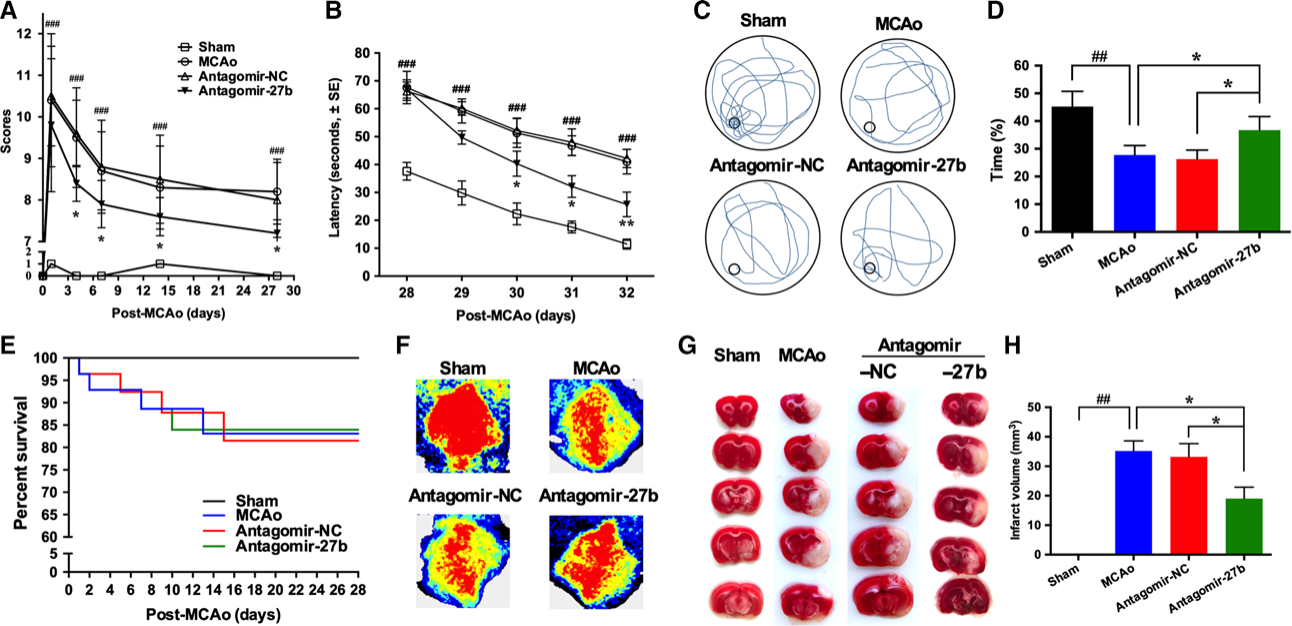
Activation of AMPK by antagomir‐27b improved neurological function after MCAo. (A) mNSS score test was performed at day 0, 1, 4, 7, 14 and 28 after MCAo. (B–D) Morris water maze test was performed from day 28 to day 32 after MCAo; average escape latency to the hidden platform is shown in (B), swim path is shown in (C), relative time of swimming in target quadrant (%) is shown in (D). (E) Survival curve of each group. (F) Representative images of CBF. (G, H) Representative images and quantification of mouse brain infraction volume from all groups after MCAo. *n* = 10/group for functional tests, *n* = 5/group for CBF test and TTC staining. The data are expressed as mean ± SEM. ^##^ *P *<* *0.01, ^###^ *P *<* *0.001 *vs* sham; *P <* *0.05, **P <* *0.01 *vs* MCAo (ANCOVA).

To verify the effects of miR‐27b inhibition in neurological outcomes after ischemic stroke. We treated mice with antagomir‐27b to silence endogenous miR‐27b after MCAo surgery, and observed neurological functional recovery. As shown in Fig. 3, the antagomir‐27b group showed a better neurological functional recovery outcome compared to the MCAo group from day 4 and this lasted through the entire study period (*P* < 0.05, Fig. 3A). From day 28, we employed the Morris water maze test to assess the spatial memory of all groups. The escape latency time for finding a hidden platform in the group that went through MCAo surgery took much longer than in the sham group. Although the escape latency time of all groups showed decreased tendency during the training period of five consecutive days, antagomir‐27b‐treated mice took a shorter time to find the platform from the third to fifth days compared with the MCAo group (*P* < 0.05, Fig. 3B). In the probe trial session, swimming time within the target quadrant was low in the MCAo group, which indicated that spatial memory of these mice was impaired after stroke. The antagomir‐27b group showed a longer swimming time in the target quadrant than the MCAo group (*P* < 0.05, Fig. 3C,D). These data strongly indicated that antagomir‐27b improved neurological function recovery of ischemic stroke mice.

During the study period, the mortality rate of the MCAo group was 17%, and treatment with antagomir‐27b showed no obvious effect (Fig. 3E). Before sacrifice, the CBF in both the contralateral and the ipsilateral half brain was measured. The ipsilateral/contralateral flow ratio of the antagomir‐27b group was significantly higher than that of the MCAo group (78.4% *vs* 62.7%, *P* < 0.05, Fig. S1). TTC staining revealed that the brain infarct volume was obviously decreased after antagomir‐27b (Fig. 3E,F).

### Antagomir‐27b enhanced neurogenesis in MCAo mice via activating AMPK

**Figure 4:**
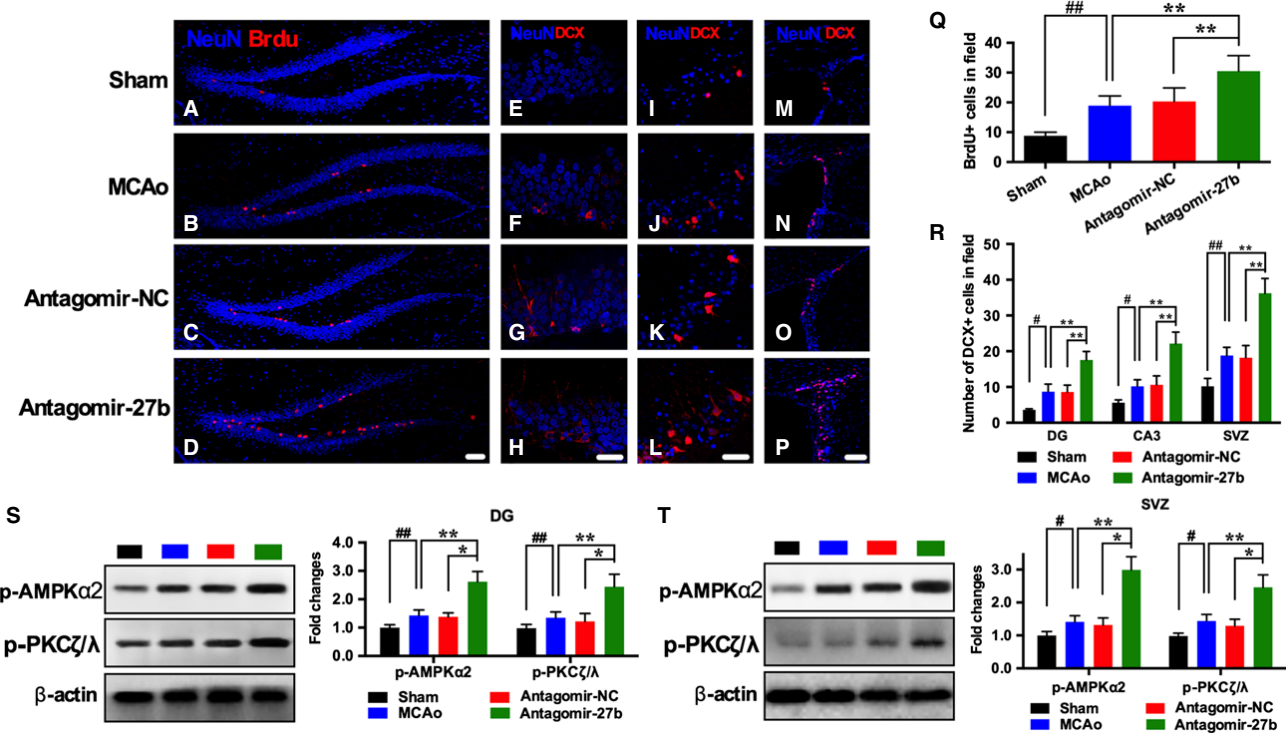
Antagomir‐27b improved neurogenesis in DG and SVZ after MCAo. (A–D, Q) Representative images and quantification of NeuN (blue) and BrdU (red) positive cells in DG. Scale bar = 100 μm. Representative images and quantification of NeuN (blue) and DCX (red) positive cells in DG (E–H, R; scale bar = 50 μm), CA3 (I–L, R; scale bar = 50 μm) and SVZ (M–P, R; scale bar = 100 μm). (S,T) Western blot analysis of p‐AMPKα2 and p‐PKCζ/λ expression and their quantitative data in DG (S) and SVZ (T). *n* = 5/group. The data are expressed as mean ± SEM. ^#^ *P *<* *0.05, ^##^ *P *<* *0.01 *vs* Sham; *P <* *0.05, **P <* *0.01 *vs* antagomir‐27b (one‐way ANOVA followed by *post hoc* Tukey's test).

To explore the mechanism beneath the therapeutic effects of antagomir‐27b on ischemic stroke mice, we further tested whether the regulation of AMPK activity would affect neurogenesis *in vivo*. Immunostaining of hippocampal sections demonstrated that cell proliferation in the subgranular zone (revealed by BrdU^+^ cell populations) showed newborn neurons were enhanced in the MCAo group. The treatment of antagomir‐27b significantly increased the number of BrdU^+^ cells by 44.5% compared with the MCAo group (Fig. 4A–D). Consistent with this, we observed an increase in cells expressing DCX, a marker of newly generated neurons, in DG (2.01‐fold, Fig. 4E–H), CA3 (2.16‐fold, Fig. 4I–L) and SVZ (1.93‐fold, Fig. 4M–P) in the antagomir‐27b‐treated group. These data indicate that neurogenesis was improved and this process could at least partially explain the improvement of neurological function in antagomir‐27b group. Western blotting demonstrated the expression of p‐AMPKα2 and p‐PKCζ/λ was significantly enhanced in DG and SVZ from the antagomir‐27b group (Fig. 4S,T).

## Discussion

In the present study, we report that the inhibition of miR‐27b improved neurogenesis in DG and SVZ via activating AMPK, and enhanced post‐stroke recovery in a mouse MCAo model, which may become a potential therapeutic option for ischemic stroke.

Ischemic stroke, accounting for ~ 80% of stroke, leads to a high mortality rate and causes irreversible disability. Nowadays, the only FDA‐approved cure, recombinant tissue plasminogen activator, has a narrow therapeutic window, which means that only ~ 4% patients receive the treatment in time 38. Owing to their high metabolism rate, high oxygen consumption and low energy storage, neurons are vulnerable to damage under conditions of ischemia and hypoxia. AMPK is a known as a sensor and regulator of energy balance. When cellular energy supply is low, as signaled by increasing AMP/ATP, AMPK is activated and regulates metabolism by directly targeting metabolic enzymes and by regulating gene transcription.

Whether AMPK plays a beneficial or a detrimental role in ischemic brain has engendered considerable controversy. It is newly recognized that AMPK activation can lead to different outcomes depending on the physiological status of the organism, and AMPK activation can protect the heart, endothelial cells, and smooth muscle cells under certain stress conditions 39, 40. Other groups reported that, short‐term AMPK activation improved cell survival since it induced nuclear translocation of forkhead box O3 (FOXO3), while long‐term of AMPK activation leads to cell death via stimulating a second activation step of FOXO3 and increasing Bim expression 41. In our study, we confirmed miR‐27b directly targets AMPK mRNA. When expression of miR‐27b was inhibited, AMPK activity was stimulated in a mild and gradual manner, without affecting the neuronal survival in the hypoxic condition but improved NSC proliferation and differentiation into neurons *in vitro*.

Neurogenesis in DG and SVZ is involved in neuronal repair after ischemic stroke. Neuroblasts from the SVZ migrate to the damaged areas and serve as a replacement of the dead neurons and help the restoration of cognition and motor skills 42. Metformin, a first‐line drug prescribed for type 2 diabetes and a well‐established AMPK activator, has been proven to exert beneficial effects on stroke patients and to increase hippocampal neurogenesis in preclinical studies via the atypical protein kinase C (aPKC)‐CREB‐binding protein (CBP) pathway 43, 44, 45. However, it has not been clarified whether the direct regulation of AMPK activity by miRNAs would affect neurogenesis. Increasing evidence has suggested that miRNAs participate in the cellular response to ischemic stroke injury 46, 47, 48. In our study, inhibition of miR‐27b enhanced both AMPK and p‐AMPK expression levels, but did not change the p‐AMPK/AMPK ratio, which differed from the mechanism of AICAR or metformin. To repair stroke‐induced cell damage, a series of energy‐consumptive pathways are activated, and an acute and high level of AMPK activation may further contribute to cell death. However, a chronic and low level of AMPK activation might accommodate the brain to metabolic stress and regulate related signaling pathways. In the present study, depressed miR‐27b expression improved stroke outcome and spatial memory, and enhanced neurogenesis in DG and SVZ. In both area, the aPKC‐CBP pathway was activated, which may explain the newborn neuron promotion, but the mechanism beneath this still needs to be explored.

In conclusion, we reported for the first time that a mild activation of AMPK by anti‐miR‐27b induced neurogenesis and promoted post‐stroke recovery. This study provides a strong experimental basis for the use of AMPK activator after ischemic stroke, which may become a potential therapeutic option.

## Conflict of interest

The authors declare no conflict of interest.

## Author contributions

ZW did the experiments design and wrote the final draft of manuscript; YY did *in vitro* experiments and wrote part of the manuscript draft; ZZ did the animal surgery and treatments; KD did functional tests, immunostaining and image analysis.

## Supporting information


**Fig. S1.** Antagomir‐27b improved CBF after MCAo. Quantitative data of CBF on day 28 after MCAo. The data are expressed as mean ± SEM. *n* = 5/group. ^###^ *P *<* *0.001 *vs* Sham; *P <* *0.05 *vs* antagomir‐27b (one‐way ANOVA followed by *post hoc* Tukey's test).
**Fig. S2.** Antagomir‐27b activated the expression of p‐AMPK *in vivo*. (A,B) Time course of p‐AMPKα2 expression in SVZ after one‐time injection of antagomir‐27b. *n* = 3/time point, mean ± SEM. *P <* *0.05, **P <* *0.01 compared with the non‐treated time point (one‐way ANOVA followed by *post hoc* Tukey's test). (C–F) Western blot analysis of p‐AMPKα2 and AMPKα2 expression and their quantitative data in cortex (C,D) and striatum (E,F). *n* = 5/group. The data are expressed as mean ± SEM. ^#^ *P *<* *0.05, ^##^ *P *<* *0.01 compared with Sham group; *P <* *0.05, **P <* *0.01 *vs* antagomir‐27b (one‐way ANOVA followed by *post hoc* Tukey's test).
