# Supplementary material for: Inhibition of miRNA‐27b enhances neurogenesis via AMPK activation in a mouse ischemic stroke model
Source: FEBS Open Bio. 2019 Apr 11;9(5):859–69. doi: 10.1002/2211-5463.12614 (PMC6487723; doi:10.1002/2211-5463.12614)
Supplement: Supplementary file 1 — Fig. S1. Antagomir‐27b improved CBF after MCAo. Quantitative data of CBF on day 28 after MCAo. The data are expressed as mean ± SEM. n = 5/group. ### P < 0.001 vs Sham; *P < 0.05 vs antagomir‐27b (one‐way ANOVA followed by post hoc Tukey's test). Fig. S2. Antagomir‐27b activated the expression of p‐AMPK in vivo. (A,B) Time course of p‐AMPKα2 expression in SVZ after one‐time injection of antagomir‐27b. n = 3/time point, mean ± SEM. *P < 0.05, **P < 0.01 compared with the non‐treated time point (one‐way ANOVA followed by post hoc Tukey's test). (C–F) Western blot analysis of p‐AMPKα2 and AMPKα2 expression and their quantitative data in cortex (C,D) and striatum (E,F). n = 5/group. The data are expressed as mean ± SEM. # P < 0.05, ## P < 0.01 compared with Sham group; *P < 0.05, **P < 0.01 vs antagomir‐27b (one‐way ANOVA followed by post hoc Tukey's test). [file FEB4-9-859-s001.docx]

Supplemental Information

Inhibition of miRNA-27b enhances Neurogenesis via AMPK activation in Mouse Ischemic Stroke Model

ZhenGang Wang, Yimei Yuan, Zhaoguang Zhang, Kuiying Ding

**
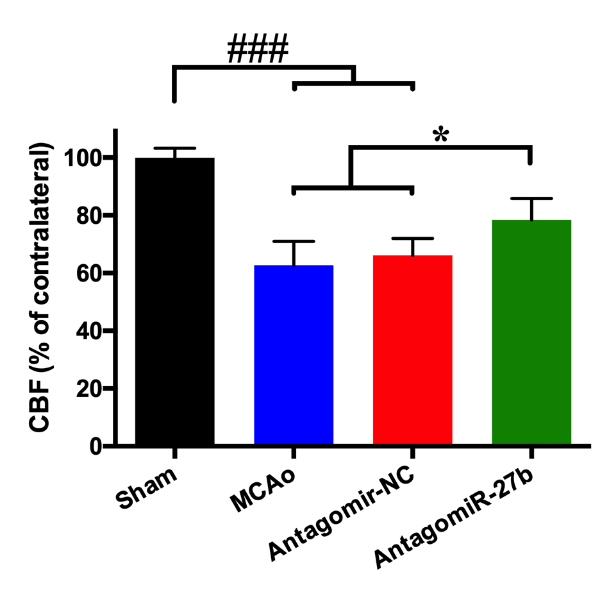
**

**Figure S1.** Quantitative data of CBF on day 28 after MCAo. n=5/group, data is expressed as mean ± SE. ### *p*<0.001 compared with Sham group; * *p*<0.05 compared with the MCAO group.

**
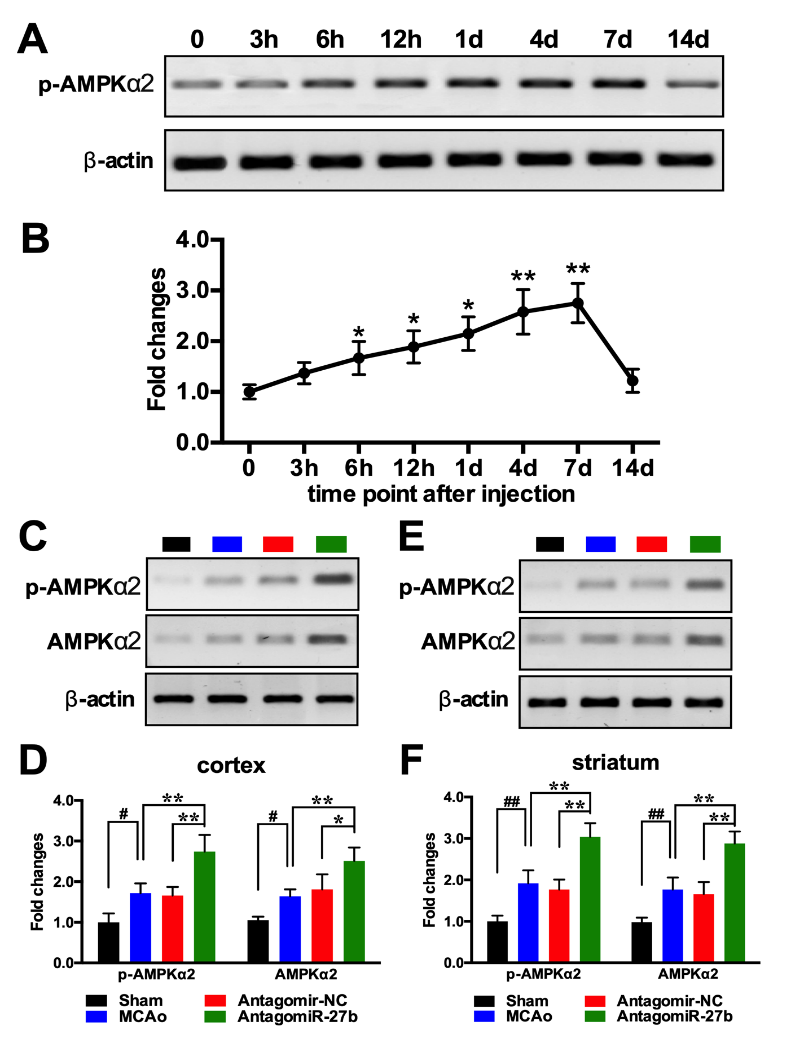
**

**Figure S2**. **A, B**. Time course of p-AMPKα2 expression in SVZ after one-time injection of Antagomir-27b, n=3/time point, mean ± SE. * *p*<0.05, ** *p*<0.01 compared with the non-treated time point; **C~F**. Western blot analysis of p-AMPKα2 and AMPKα2 expression contents and their quantitative data in cortex (**C, D**) and striatum (**E, F**). n=5/group, mean ± SE. # *p*<0.05 ## *p*<0.01 compared with Sham group; * *p*<0.05, ** *p*<0.01 compared with the MCAO group.
